# Supplementary material for: PHERI—Phage Host ExploRation Pipeline
Source: Microorganisms. 2023 May 26;11(6):1398. doi: 10.3390/microorganisms11061398 (PMC10305434; doi:10.3390/microorganisms11061398)
Supplement: Supplementary file 1 [file microorganisms-11-01398-s001.zip › microorganisms-2398512-supplementary.pdf]

Supplementary materials

PHERI—Phage Host ExploRation Pipeline

Andrej Baláž, Michal Kajsík , Jaroslav Budiš, Tomáš Szemeš, Ján Turňa

Table S1 Cluster number, position and predicted function of Dev-CS701 phage proteins suitable for decision trees of the genera *Cronobacter* and *Citrobacter*

Citrobacter **tree**			*Cronobacter* **tree**		
Position	Cluster	Present/Function	Position	Cluster	Present/Function
0	743		0	2261	No
1	170	Hinge connector of long tail fiber distal connector	1	1221	No
1	170	L-shaped tail fiber protein	2	247	Yes/Clamp loader subunit
2	918	No	3	714	No
3	966	No	4	426	Yes/putative endolysin
4	365	No	5	170	Yes/Hinge connector of long tail fiber distal connector
5	828	Unknown	5	170	Yes/L-shaped tail fiber protein
6	19	No	6	830	Yes/Peptidoglycan binding protein
7	186	Portal vertex protein	7	54	Yes/MobE endonuclease
8	23	DNA methylase	8	228	No
9	54	MobE endonuclease	9	640	No
			10	104	No
			11	679	No

The trees represents the resulting classifier for a single host genus. Each node compares a number of genes of a phage in a particular cluster and a learned threshold. If the number of genes in cluster is smaller than the threshold, the classification decision moves to the left, otherwise to the right. Upon reaching a leaf, the final decision if the phage's host belongs to the genus or not is made. The values [x, y] represent the number of phages from training set for each case at a particular node. For example, at the tree root we can see that 4706 phages from training set did not infect this particular host and 17 phages did. Gray boxes represent those phage protein sequences whose annotated function is known

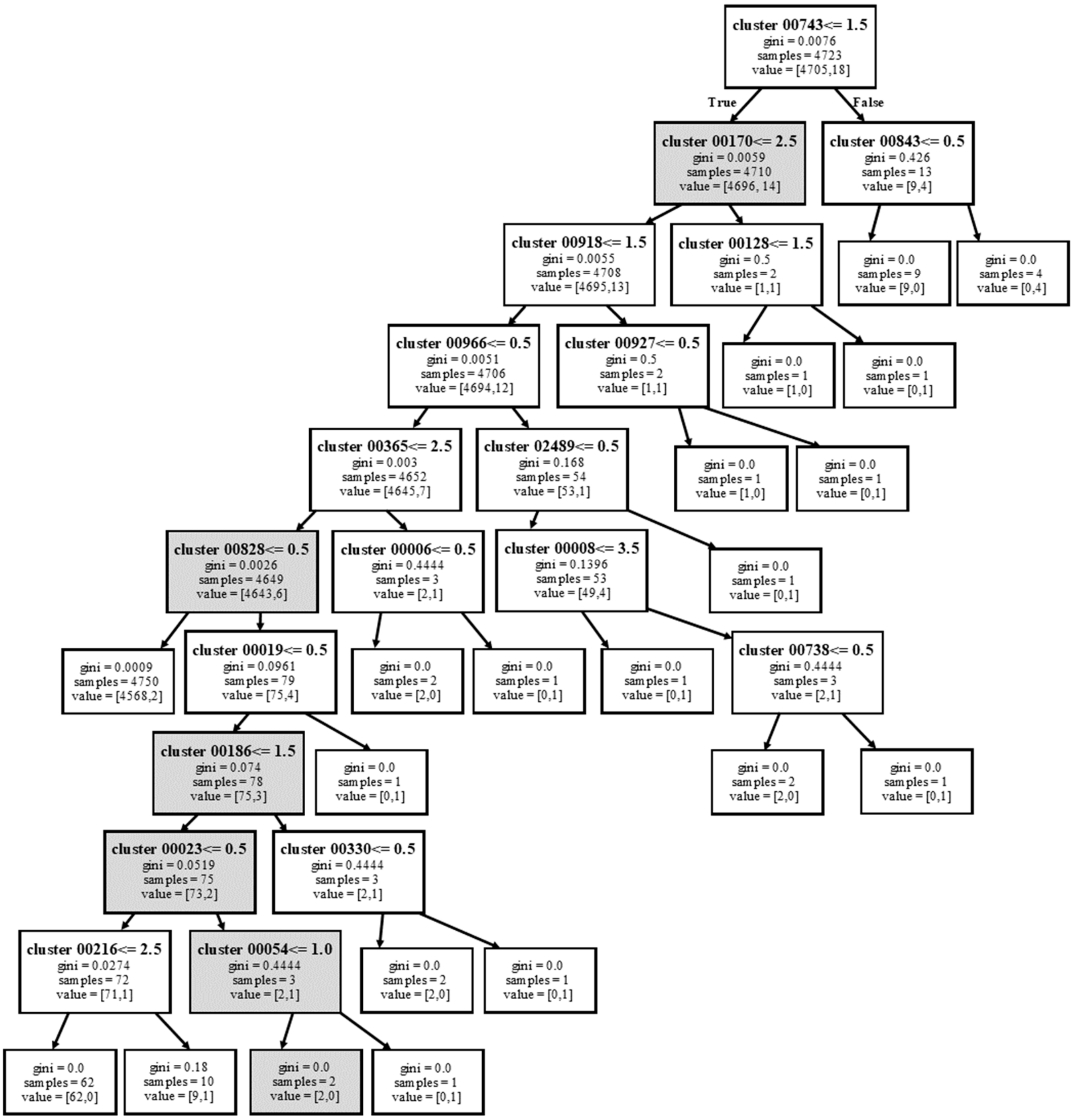

Figure S1 Citrobacter decision tree. Each position in the decision tree consists of a cluster number. The cluster represents a group of proteins with a certain similarity. It further contains a condition representing the number of proteins from a given cluster that must be present in the phage being tested. The gini coefficient is a statistical measure of distribution. The coefficient ranges from 0 to 1, with 0 representing perfect equality and 1 representing perfect inequality of proteins in the cluster. The number of samples represents how many phages meet the conditions of the previous tree levels and how this cluster divides the phages into those that meet the condition and those that do not.

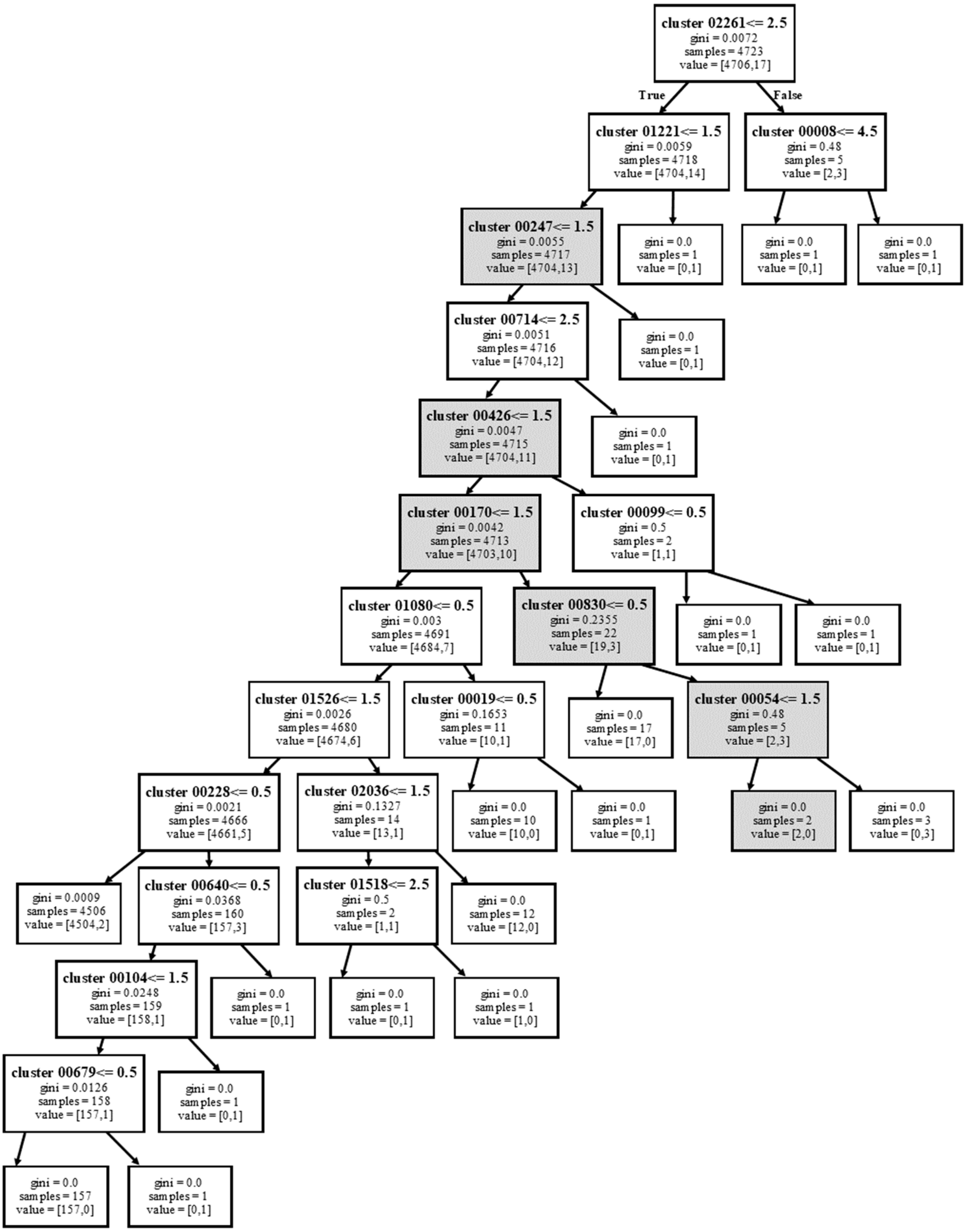

Figure S2 Cronobacter decision tree. Each position in the decision tree consists of a cluster number. The cluster represents a group of similar proteins. It further contains a condition representing the number of proteins from a given cluster that must be present in the phage being tested. The gini coefficient ranges from 0 to 1, with 0 representing perfect equality and 1 representing perfect inequality of proteins in the cluster. The number of samples represents how many phages meet the conditions of the previous tree levels and how this cluster divides the phages into those that meet the condition and those that do not
